# *Legionella pneumophila*-Derived Outer Membrane Vesicles Promote Bacterial Replication in Macrophages

**DOI:** 10.1371/journal.ppat.1005592

**Published:** 2016-04-22

**Authors:** Anna Lena Jung, Cornelia Stoiber, Christina E. Herkt, Christine Schulz, Wilhelm Bertrams, Bernd Schmeck

**Affiliations:** 1 Institute for Lung Research, Universities of Giessen and Marburg Lung Center, Philipps-University Marburg, Member of the German Center for Lung Research (DZL), Marburg, Germany; 2 Institute for Virology, Philipps-University Marburg, Marburg, Germany; 3 Department of Medicine, Pulmonary and Critical Care Medicine, University Medical Center Giessen and Marburg, Philipps-University, Member of the German Center for Lung Research (DZL), Marburg, Germany; Purdue University, UNITED STATES

## Abstract

The formation and release of outer membrane vesicles (OMVs) is a phenomenon of Gram-negative bacteria. This includes *Legionella pneumophila* (*L*. *pneumophila*), a causative agent of severe pneumonia. Upon its transmission into the lung, *L*. *pneumophila* primarily infects and replicates within macrophages. Here, we analyzed the influence of *L*. *pneumophila* OMVs on macrophages. To this end, differentiated THP-1 cells were incubated with increasing doses of *Legionella* OMVs, leading to a TLR2-dependent classical activation of macrophages with the release of pro-inflammatory cytokines. Inhibition of TLR2 and NF-κB signaling reduced the induction of pro-inflammatory cytokines. Furthermore, treatment of THP-1 cells with OMVs prior to infection reduced replication of *L*. *pneumophila* in THP-1 cells. Blocking of TLR2 activation or heat denaturation of OMVs restored bacterial replication in the first 24 h of infection. With prolonged infection-time, OMV pre-treated macrophages became more permissive for bacterial replication than untreated cells and showed increased numbers of *Legionella*-containing vacuoles and reduced pro-inflammatory cytokine induction. Additionally, miRNA-146a was found to be transcriptionally induced by OMVs and to facilitate bacterial replication. Accordingly, IRAK-1, one of miRNA-146a’s targets, showed prolonged activation-dependent degradation, which rendered THP-1 cells more permissive for *Legionella* replication. In conclusion, *L*. *pneumophila* OMVs are initially potent pro-inflammatory stimulators of macrophages, acting via TLR2, IRAK-1, and NF-κB, while at later time points, OMVs facilitate *L*. *pneumophila* replication by miR-146a-dependent IRAK-1 suppression. OMVs might thereby promote spreading of *L*. *pneumophila* in the host.

## Author Summary

Of all intracellular pathogens, *Legionella pneumophila* show the highest genetic adaptation to the host. They are important human pathogens causing mainly pneumonia, but are also highly successful pathogens of water amoeba. To manipulate host cells for optimal intracellular replication, they express several hundred specific virulence factors, which they apply to their host cells by two different secretion systems. Another intriguing avenue that leads to the transfer of virulence factors to host cells—even over distance—is the transport via outer membrane vesicles (OMV) that are released by *Legionella*. We report here that after exposure to these vesicles, macrophages become permissive hosts for bacterial replication incapable of efficient pathogen defense. In addition, markers of inflammation that are induced by OMV pre-treatment do not further increase upon subsequent bacterial infection. This goes along with improved survival of infected macrophages despite the increased intracellular bacterial growth. We could attribute this effect to TLR- and NF-κB-dependent mechanisms of microRNA-146a induction and prolonged IRAK-1 depletion.

## Introduction

Bacteria have developed numerous strategies to deliver virulence factors into their eukaryotic host cells. In close proximity, the transfer of virulence factors can take place by direct translocation into the host cytosol. Distant cells can be reached by the secretion of soluble proteases, lipases or toxins to the extracellular environment [1]. Additionally, Gram-negative bacteria developed the strategy of outer membrane vesicle (OMV) formation. OMVs are small, spheroid membrane vesicles of 10–300 nm in diameter, secreted during all phases of growth as well as in a variety of growth environments (liquid culture, solid culture, biofilms) [2, 3]. They transport diverse virulence factors, including proteins, adhesins, toxins and enzymes as well as non-protein antigens such as lipopolysaccharide (LPS), which is present on the outer leaflet of the OMV membrane [4]. They serve as a means of communication among bacteria, but can also be recognized and taken up by eukaryotic cells [4]. OMVs may influence the course of infection and the host immune response by presenting pathogen-associated molecular patters (PAMPs) and antigens to their respective host receptors [5]. For example OMVs derived from *Clostridium perfringens* induce cytokine secretion in macrophages, *Borellia burgdorferi* OMVs activate B cells, and vesicles secreted by *Helicobacter pylori* act on gastric epithelial cells [6–8]. In addition, OMVs transport active virulence factors of Gram-negative bacteria which gain access to the extracellular environment and can act over long distances, since the vesicular membrane protects the luminal cargo from extracellular host proteases and facilitates penetration into tissue [9–11]. OMVs have been found not only in close proximity to the site of bacterial colonization, but also in body fluids and distant organs [12]. Furthermore, they can mediate bacterial binding and invasion into host cells and cause cytotoxicity [4].

*Legionella pneumophila* (*L*. *pneumophila*) is a Gram-negative bacterium that replicates in freshwater amoebae [13]. When it enters the human lung, it primarily infects alveolar macrophages and can cause legionnaires’ disease, an acute fibrinopurulent pneumonia [14]. Macrophages have been described to respond to *L*. *pneumophila* infection by up-regulation of TNF-α, IL-6 and IL-1β [15]. These factors potently contribute to the establishment of a pro-inflammatory activation state, which is commonly referred to as classical macrophage activation (M1). In contrast, alternatively activated macrophages (M2), as characterized *e*.*g*. by CD206 (MRC1), do not show increased bactericidal potential. Upon infection, macrophages establish a transient activation state in the spectrum between these two canonical states, and this balance is decisive for the disposal of intracellular bacteria. *L*. *pneumophila* manipulates the host by secreting effector proteins into the cytoplasm via its type IV secretion system, leading to the blockage of phagosome-lysosome fusion, thus enabling *L*. *pneumophila* to recruit host organelles and to form its replication niche, the *Legionella* containing vacuole (LCV) [16, 17]. The inhibition of phagosome-lysosome fusion is achieved by effector protein secretion, and also by secretion of OMVs [18]. A proteomic analysis of *L*. *pneumophila* OMVs revealed about 70 proteins, many of them exclusively secreted via OMVs and associated with virulence function, *e*.*g*. persistence and spreading in the lung (fliC [19]), invasion (IcmK [20]) or intracellular survival and replication (ProA1 [21]). Additionally, *L*. *pneumophila* OMVs display proteolytic and lipolytic activity *in vitro* [22]. Experiments with human lung tissue explants (HLTE) demonstrated that OMVs can cause tissue damage [23]. Immune histological analysis of OMV-treated HLTE showed that OMVs were mainly bound to alveolar macrophages [24]. Since it is known that *L*. *pneumophila* OMVs are pro-inflammatory activators of macrophages and epithelial cells [22, 23], we aimed to analyze the impact of *L*. *pneumophila* OMVs on macrophage activation and their influence on a subsequent infection with *L*. *pneumophila*. As the innate immune response largely depends on the recognition of PAMPs, *e*.*g*. LPS, in their natural context, we speculate that *L*. *pneumophila* OMVs could have immunomodulatory functions, as already described for *Brucella abortus* OMVs [25] and OMVs derived from *Porphyromonas gingivalis* [26]. We investigated for the first time the influence of *L*. *pneumophila* OMV pre-treatment on a subsequent infection with *L*. *pneumophila* and the role of PAMP receptors and associated signaling pathways in OMV sensing.

## Results

### Pro-inflammatory activation of macrophages by *L*. *pneumophila* OMVs

**Fig 1:**
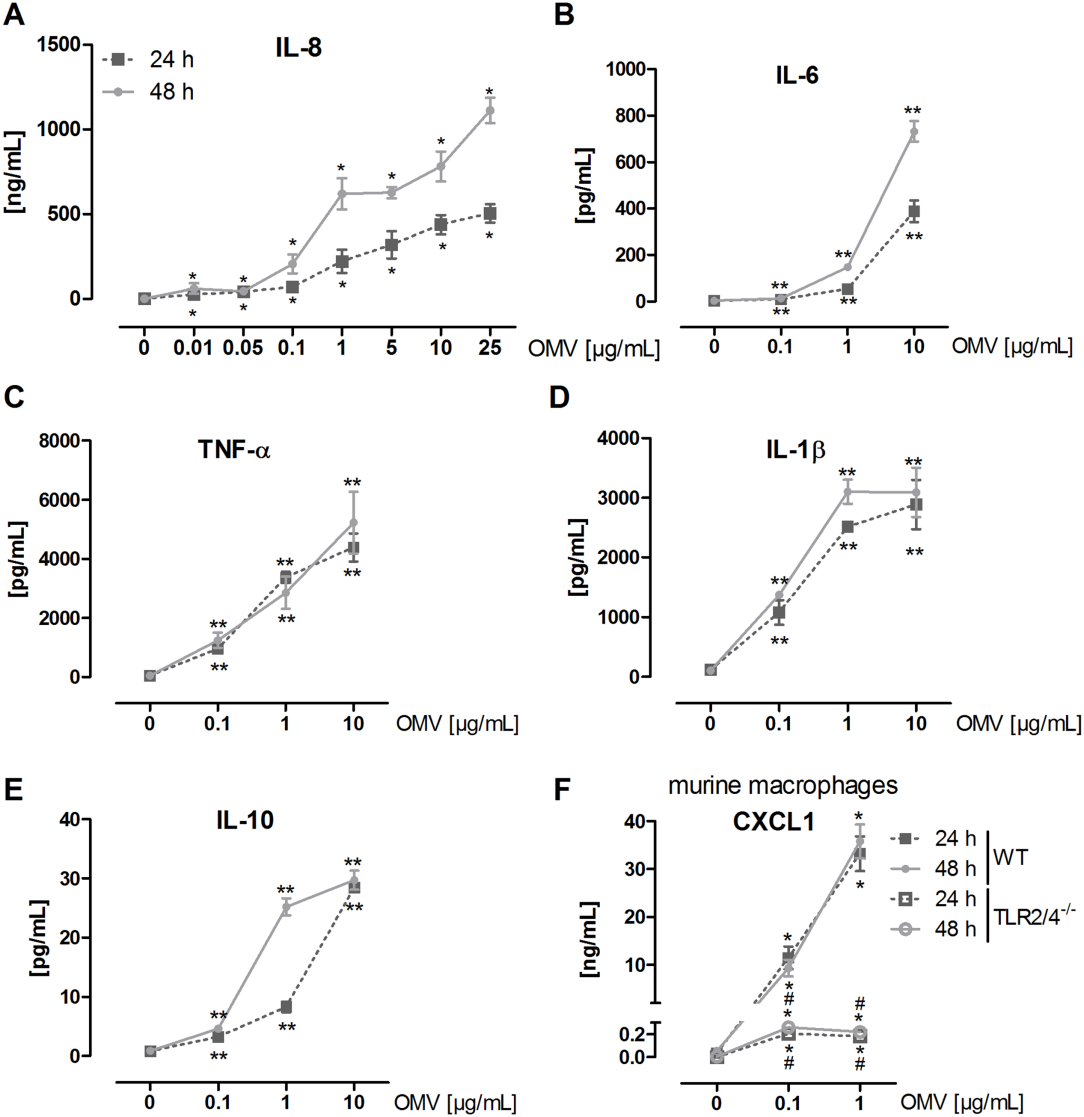
*L*. *pneumophila* OMVs induce cytokine secretion in macrophages. (A) THP-1 cells were incubated with rising amounts of OMVs (0.01, 0.05, 0.1, 1, 5, 10, 25 μg/mL) for 24 or 48 h, respectively, or left untreated for control. Supernatant was collected for IL-8 ELISA. (B-E) THP-1 cells were incubated with rising amounts of OMVs (0.1, 1, 10 μg/mL) for 24 or 48 h, respectively, or left untreated for control. Supernatant was collected for magnetic multiplex ELISA. Results for IL-6 (B), TNF-α (C), IL-1β (D), and IL-10 (E) are shown. (F) mBMDM (wildtype (WT) and TLR2/4^-/-^) were stimulated with OMVs (0.1, 1 μg/mL) for 24 or 48 h, respectively, or left untreated for control. Supernatant was collected for CXCL1 ELISA. Mean values of three independent experiments are shown. Statistics: Mann-Whitney test; *p<0.05 and **p<0.01 compared to corresponding control. #p<0.05 compared to equally treated WT sample.

As macrophages are the main target of *L*. *pneumophila* infection and replication [14], we tested the effect of *L*. *pneumophila* OMVs on macrophages. We incubated PMA-differentiated macrophage-like THP-1 cells with increasing doses of OMVs (0.01–25 μg/mL according to the protein concentration) for 24 and 48 h, respectively. The lowest OMV dose (0.01 μg/mL) was sufficient to induce a significant pro-inflammatory IL-8 release in THP-1 cells (Fig 1A) that increased time- and dose-dependently. Moreover, we observed a time- and dose-dependent release of IL-6 (Fig 1B) and IL-10 (Fig 1E) and a dose-dependent release of TNF-α (Fig 1C) and IL-1β (Fig 1D).

To gain further insight into the OMV amounts present under infection conditions, we generated bacteria-free cell culture supernatant of infected THP-1 cells (MOI 0.5, 24 h) by sterile filtration. The remaining membrane bodies were pelleted by differential centrifugation and probed for their LPS content. We detected LPS amounts which equal 0.04–0.08 μg of free OMVs. Given the fact that *L*. *pneumophila* OMVs are rapidly internalized by macrophages [24] and that we could only measure free OMVs after the incubation time, 0.1, 1, and 10 μg/mL OMVs were used for the following experiments.

No decrease in cell viability was observed by MTT assay for up to 96 h of OMV incubation (S1 Fig) in accordance to previous studies [22, 24].

Since macrophages sense *L*. *pneumophila* via LPS on the bacterial surface mainly by TLR2 [27, 28], we hypothesized that OMVs might stimulate macrophages in a similar way. We used murine bone marrow-derived macrophages (mBMDM) from wildytpe and TLR2/4^-/-^ mice, which were treated with 0.1 or 1 μg/mL OMVs for 24 and 48 h, respectively. CXCL1, the murine functional homologue of IL-8, served as a measure for pro-inflammatory activation of mBMDM. Wildtype mBMDM secreted significant amounts of CXCL1 dose-dependently when incubated with OMVs (Fig 1F). Contrary to wildtype cells, TLR2/4^-/-^ cells showed a rigorously reduced response to OMVs, as CXCL1 secretion was only slightly above the detection limit (0.2 ng/mL for both OMV concentrations). These results demonstrate that *L*. *pneumophila* OMVs are pro-inflammatory stimulators of macrophages and that macrophage activation occurs in a TLR2-dependent manner.

### *L*. *pneumophila* OMVs alter bacterial replication in macrophages

**Fig 2:**
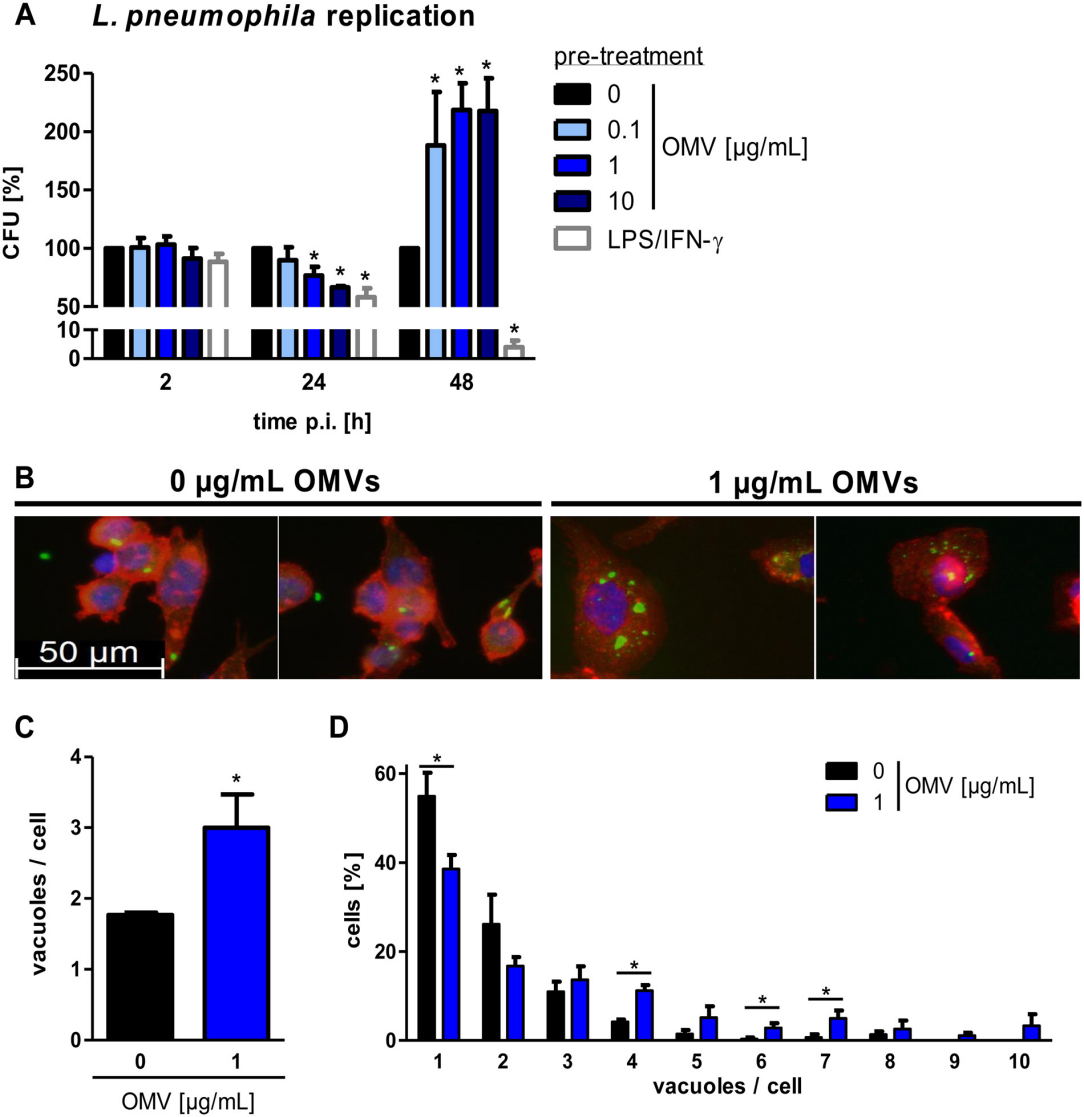
*L*. *pneumophila* OMVs alter bacterial replication and increase the vacuole amount per cell. (A) THP-1 cells were pre-incubated for 20 h with different doses of OMVs (0.1, 1, 10 μg/mL) and were then infected with *L*. *pneumophila* (multiplicity of infection (MOI) of 0.5). Pre-stimulation with LPS (200 ng/mL) in combination with IFN-γ (200 ng/mL) served as a positive control for classically activated macrophages (M1). Bacterial replication after OMV pre-stimulation was determined by CFU assay. Cells were lysed after the indicated time points. Results are depicted relative to CFU count of not pre-treated (but infected) cells at every time point. (B) THP-1 cells were pre-treated with OMVs (1 μg/mL) for 20 h or left untreated before infection with *L*. *pneumophila* (MOI 0.5) for 48 h. Cells were fixed and stained with an α-LPS antibody (green), phalloidin (red), and DAPI (blue). One representative out of three independent experiments is shown. (C) Quantification of the number of vacuoles per cells of more than 70 cells per condition. Data are shown as mean values of three independent experiments. (D) Quantification of the distribution of vacuoles per cells. Mean values of three independent experiments are shown. Statistics: Mann-Whitney test; *p<0.05 compared to 0 μg/mL OMVs.

We investigated whether cellular contact with *L*. *pneumophila* OMVs has an impact on a following encounter with *L*. *pneumophila*. To this end, cells were pre-incubated with OMVs for 20 h, as they already responded with a pro-inflammatory response at this time point, and then infected with *L*. *pneumophila*. Cells were lysed at different time points to monitor bacterial uptake (2 h post infection (p.i.)) and replication (24 and 48 h p.i.) by CFU (colony forming unit) count. Infected cells without OMV pre-treatment served as a reference, and *L*. *pneumophila* replication in not pre-treated THP-1 cells is shown in S2A Fig. LPS/IFN-γ stimulation of THP-1 cells was used to induce a classical (M1) phenotype, which enables macrophages to efficiently kill *L*. *pneumophila* [29]. Lysis of cells 2 h p.i. did not reveal differences in bacterial uptake capacity in any of the tested conditions (Fig 2A and S2B Fig). Bacterial replication was significantly reduced at 24 h p.i. by 42% after LPS/IFN-γ pre-treatment. OMV pre-incubation showed similar tendencies; 0.1 μg/mL OMVs only modestly reduced *L*. *pneumophila* replication (10% reduction), whereas 1 μg/mL and 10 μg/mL of OMVs significantly reduced the bacterial load 24 h p.i. (23% and 33% reduction). At 48 h p.i., LPS/IFN-γ treated cells had progressively killed *L*. *pneumophila* (96% reduction). In contrast to LPS/IFN-γ, OMV pre-treatment had an enhancing effect on bacterial load (0.1 μg/mL: 88%, 1 μg/mL: 119%, 10 μg/mL: 118% more as compared to control). Additionally, we tested whether this mechanism also holds true with an *L*. *pneumophila* mutant which is not able to establish its replication vacuole in macrophages as it lacks a functional dot/icm system [17, 30]. To this extent, THP-1 cells were pre-stimulated with OMVs from WT *L*. *pneumophila* and infected with *L*. *pneumophila* ΔdotA mutant. Bacterial replication was analyzed by CFU assay. Interestingly, the highest dose of OMVs could enhance the replication of *L*. *pneumophila* ΔdotA by a factor of more than 14 at 48 h p.i. (S2C Fig).

Overall, pre-incubation of macrophages with OMVs leads to an altered bacterial replication.

### OMVs enhance the intracellular vacuole amount in a subsequent infection with *L*. *pneumophila*

As we detected a doubling in *L*. *pneumophila* replication after OMV pre-treatment, we aimed to gain insight into the fate of intracellular *L*. *pneumophila*. Immunofluorescence microscopy analysis was performed with not pre-treated and OMV pre-treated THP-1 cells which were subsequently infected with *L*. *pneumophila* for 48 h. After exposure of THP-1 cells to OMVs, an increased maximal cell diameter was observed (33.2 μm vs 19.1 μm). Not pre-treated THP-1 cells had one or two vacuoles per cell (Fig 2B and 2C), while in THP-1 cells with OMV pre-treatment, several LPS positive vacuoles were detected within the cytoplasm. Eighty percent of the not pre-treated cells had one or two vacuoles per cell. In contrast, only 56% of OMV pre-treated cells had one or two vacuoles (Fig 2D).

Contact with OMVs before an infection with *L*. *pneumophila* seems to increase the vacuole amount per cell in macrophages.

### OMVs render macrophages less responsive to an infection with *L*. *pneumophila*

**Fig 3:**
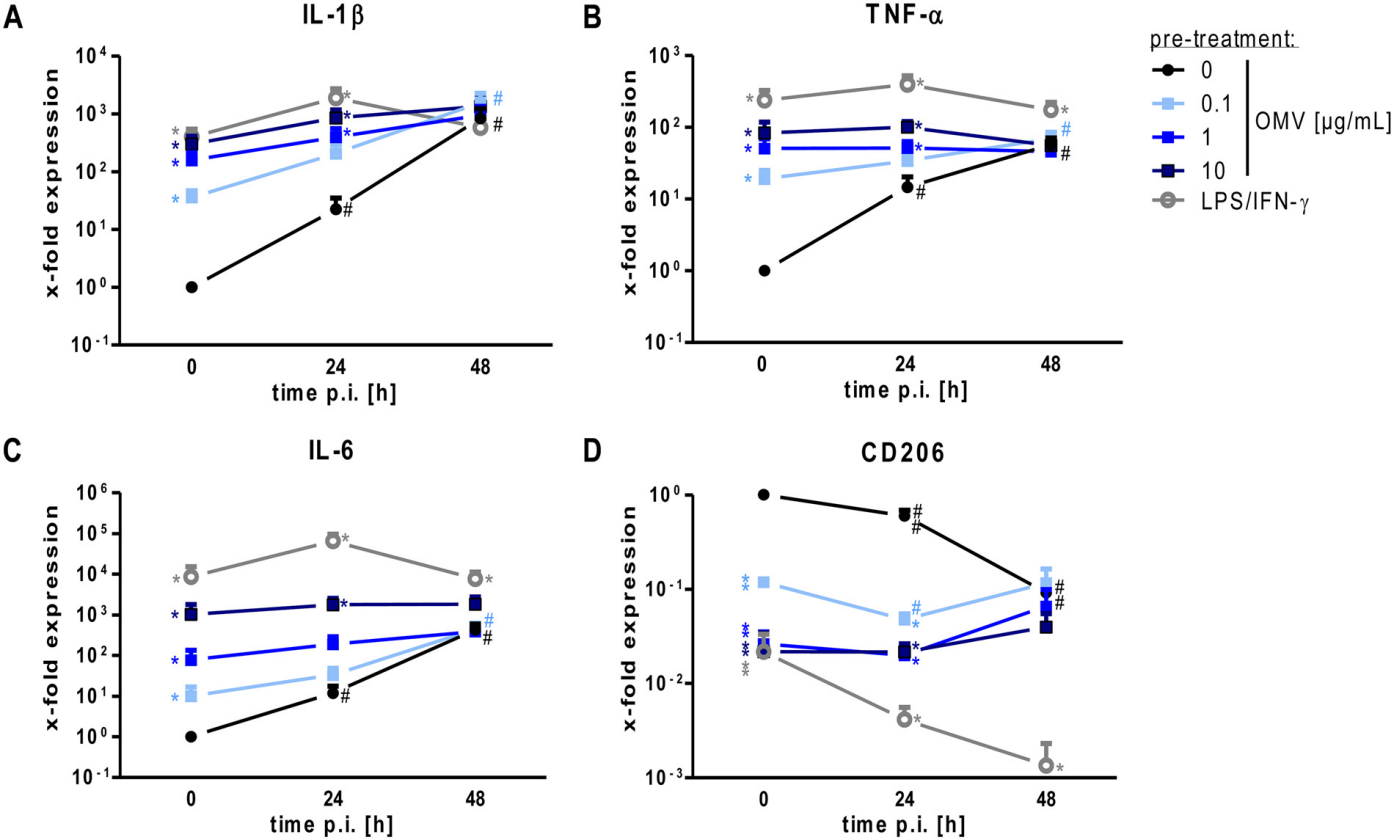
OMVs reduce responsiveness of macrophages to *L*. *pneumophila* infection. (A-D) THP-1 cells treated as described in Fig 2A and RNA samples were taken at the time of infection (0 h) or 24 and 48 h post infection (p.i.). qPCR was performed for markers of classically activated macrophages (A: IL-1β, B: TNF-α, C: IL-6) and alternatively activated macrophages (D: CD206). Results are normalized to untreated control cells at every time point. Mean values of three independent experiments are shown. Statistics: Mann-Whitney test; * p<0.05 compared to 0 μg/mL OMVs. # p<0.05 compared to equally treated 0 h sample.

We further examined the expression of established markers of classically activated macrophages (M1; IL-1β, TNF-α, and IL-6) and alternatively activated macrophages (M2; CD206) [31, 32]. After infection of not pre-treated THP-1 cells, an increase in IL-1β expression was observed (Fig 3A). In contrast, while both pre-treatments (OMVs or LPS/IFN-γ) increased basal levels of IL-1β mRNA, they inhibited a further transcriptional induction above the initially induced level. Only the lowest dose of OMVs (0.1 μg/mL) that triggered a weak induction of IL-1β transcript allowed a minor additional increase after 48 h of infection. Similar results were obtained for TNF-α (Fig 3B) and IL-6 (Fig 3C). CD206, a marker for alternatively activated macrophages, was down-regulated after *L*. *pneumophila* infection of not pre-treated cells (Fig 3D). OMV pre-treatment resulted in a significantly reduced expression of CD206, which was comparable to infected and not pre-treated cells at 48 h p.i.. These experiments demonstrate a reduced M1 response of macrophages to an infection with *L*. *pneumophila* when pre-incubated with OMVs in comparison to naïve macrophages.

### LPS is important for the response of macrophages to OMVs

We then aimed to identify the OMV component mediating the observed differences in bacterial replication and M1 activation. OMVs were incubated for 5 min at 60°C, denaturing proteins but preserving OMV shape and membrane integrity [33]. Cells were pre-treated with heated OMVs and infected with *L*. *pneumophila*, and bacterial replication was determined by CFU assay. Here, the effect of 1 μg/mL OMVs was compared to 1 μg/mL heated OMVs. Interestingly, the heated OMVs did not significantly alter the bacterial replication at 24 h p.i. in comparison to not pre-treated cells (8% reduction; S3A Fig). At 48 h p.i., cells pre-treated with heat-denaturated OMVs were equally permissive to *L*. *pneumophila* replication as cells pre-treated with non-heated OMVs.

OMVs display LPS on their surface, which is a potent activator of macrophages. The LPS concentration of the OMV preparations was determined by Limulus amebocyte lysate test. OMVs contained 0.22 μg LPS per 1 μg protein. *L*. *pneumophila* has a unique LPS structure, which is sensed via TLR2 on the cell surface [27, 34]. Thus, a lipoteichoic acid and lipoprotein containing cell wall preparation (LTA), activating TLR2, or a *Salmonella minnesota* LPS preparation, activating TLR4, were used for alternative pre-treatment of THP-1 cells to test whether TLR activation alone is able to alter the response of macrophages to an infection with *L*. *pneumophila*. The bacterial load was significantly reduced by TLR2 or TLR4 activation before the infection (24 h p.i.: 19% or 12% reduction; 48 h p.i.: 39% or 60% reduction; S3B Fig). Furthermore, a TLR2 blocking antibody was used alone or in combination with OMVs to analyze the influence of TLR2 signaling—presumably activated by LPS—in the response of macrophages to *L*. *pneumophila* OMVs. Blockage of TLR2 signaling before infection did not significantly alter *L*. *pneumophila* replication, as determined by CFU assay. The combination of OMVs with the blocking antibody led to significantly more bacteria at 24 h p.i. (22% increase), but did not alter the bacterial load at 48 h p.i.. Incubation with a control antibody in combination with OMVs did not reveal significant differences.

To validate blocking efficiency of the TLR2 antibody we analyzed expression of macrophage activation markers. The combination of OMVs with the TLR2 blocking antibody led to a reduced mRNA induction of the M1 markers IL-1β (S3C Fig), TNA-α (S3D Fig) and IL-6 (S3E Fig), whereas the expression of the M2 marker CD206 was increased (S3F Fig).

Furthermore, *L*. *pneumophila* replication after OMV pre-incubation was analyzed in mBMDM from different genetic backgrounds. In WT mBMDM, OMV pre-treatment before infection induced the bacterial replication by a factor of 15 (S3G Fig). In mBMDM lacking TLR2 receptor bacterial replication was significantly reduced in comparison to WT cells. Additionally, we used TRIF/MyD88 double knockouts to block any TLR signaling in CFU assays. TRIF/MyD88^-/-^ cells showed a response to OMV pre-treatment similar to TLR2^-/-^ cells, as the replication was 7.5 fold reduced compared to WT mBMDM.

Taken together, inhibition of TLR2 activation blocked the initial bacterial killing in the macrophages, and bacterial replication was reduced at later time points. Therefore, TLR2 activation seems to be of equal importance for the initial stimulation of macrophages and the bacterial replication.

### OMV membrane integrity is essential for the macrophage response

Since it has been shown for *L*. *pneumophila* OMVs that they may contain fliC [22], which is able to induce pro-inflammatory cell activation via TLR5 [35], we tested whether flagellin was inducing the observed effect. For this, cells were pre-exposed to OMVs obtained from ΔflaA *L*. *pneumophila* which were generated as the wildtype OMVs, but no differences could be observed in comparison to OMVs from wildtype *L*. *pneumophila* in the ability to render THP-1 cells more permissive for bacterial replication (S4A Fig).

As OMVs do not only contain LPS and proteins but also nucleic acids [36, 37], we assessed their influence on *L*. *pneumophila* replication in macrophages in more detail. OMVs were treated with RNases (RNase A and RNase III) or DNase in the presence or absence of Triton X-100. In the following CFU assay, disruption of OMV membrane prevented the increase in bacterial replication in THP-1 macrophages (S4B Fig). At 24 h p.i., Triton X-100 treatment of OMVs did not change bacterial replication in comparison to untreated control cells. This correlation was still observed upon 48 h p.i. while untreated OMVs increased the replication 4 fold. The exposure of OMVs to RNases or DNase alone did not alter *L*. *pneumophila* replication. Addition of Triton X-100 to RNases/DNases reduced *L*. *pneumophila* replication comparable to Triton X-100 alone.

Therefore we conclude that OMVs membrane integrity is essential for the response of macrophages to a following infection with *L*. *pneumophila*.

### NF-κB is involved in the macrophage response to OMVs from *L*. *pneumophila*

A key molecule downstream of TLR signaling is the transcription factor NF-κB [38]. As NF-κB activation is rapidly seen after *L*. *pneumophila* infection [39], we asked whether NF-κB is equally important in the OMV context. Cell fractionation experiments were performed after short time OMV incubation (30 min) and we could demonstrate that NF-κB subunit p65 is rapidly shuttling into the nucleus after OMV stimulation of THP-1 cells (S5 Fig).

**Fig 4:**
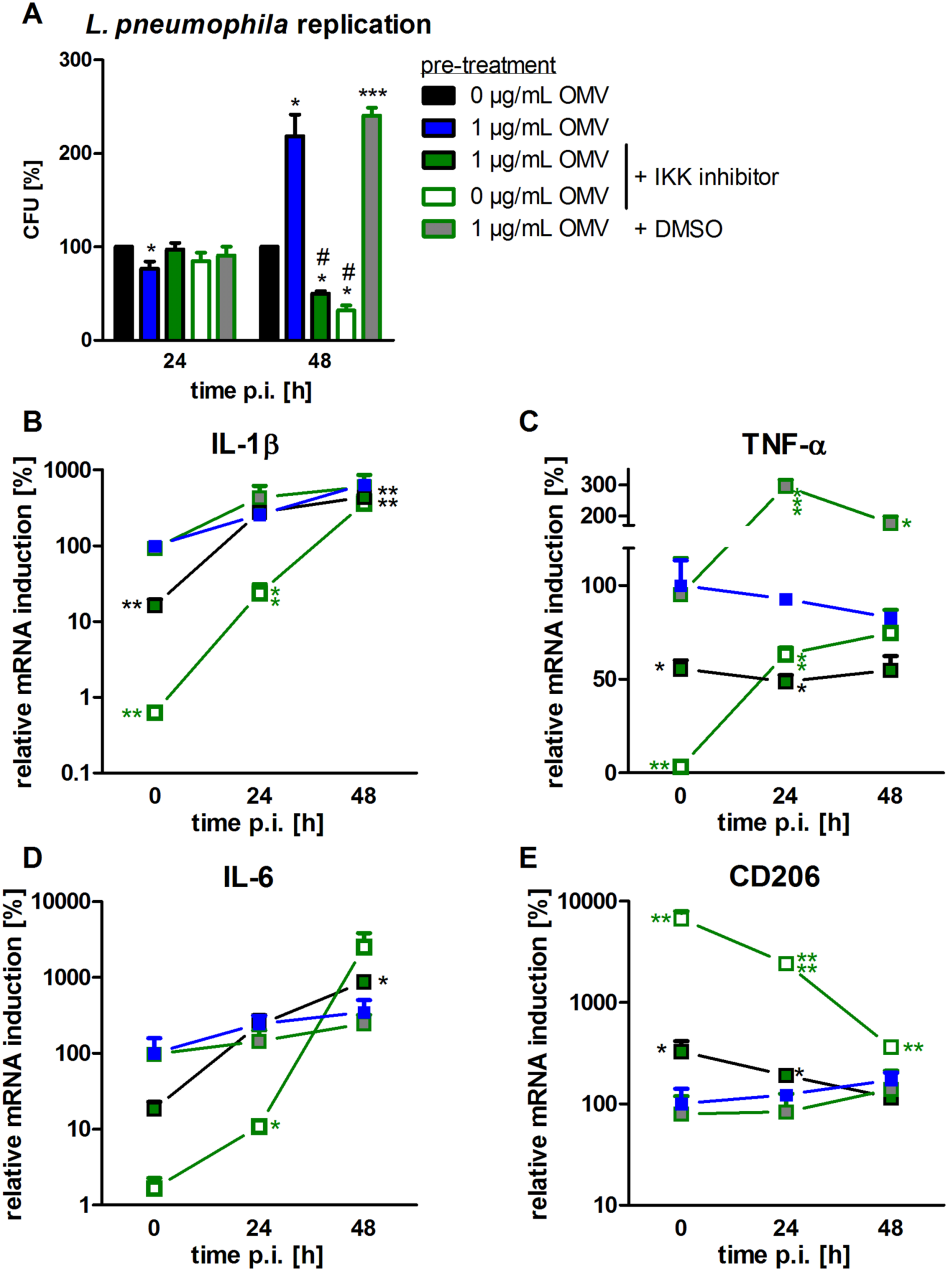
Blocking of NF-κB signaling prior to OMV treatment reduces bacterial replication and M1 activation. THP-1 cells were pre-incubated with IKK inhibitor (1 μM) 90 min before OMV stimulation (1 μg/mL). DMSO was used as a solvent control. After 20 h of OMV incubation, cells were infected with *L*. *pneumophila* (MOI 0.5) and lysed for CFU 24 and 48 h p.i.. Results are depicted relative to CFU count of not pre-treated (but infected) cells (A). RNA samples were taken at the time point of infection (0 h) or 24 and 48 h p.i.. qPCR was performed for IL-1β (B), TNF-α (C), IL-6 (D), and CD206 (E). Results are calculated relative to the time point of infection (0 h) with 1 μg/mL OMV treatment. Mean values of three independent experiments are shown. Symbol key is used for all parts of the figure. Statistics: A: Mann-Whitney test; *p<0.05 compared to 0 μg/mL OMVs. #p<0.05 compared to 1 μg/mL OMVs. B-E: Mann-Whitney test; *p<0.05, **p<0.01, ***p<0.001, ****p<0.0001 compared to 1 μg/mL OMV treatment.

We then inhibited the IKK complex with a small molecule inhibitor, which was used alone or in combination with OMV pre-treatment before THP-1 cells were infected with *L*. *pneumophila*, after testing that the IKK inhibitor does not reduce the cell viability (S6 Fig). CFU count revealed that NF-κB inhibition did not affect the early phase of *L*. *pneumophila* replication. Interestingly, it influenced the long-term replication, as observed by a reduction in bacterial replication by 68% at 48 h p.i. (Fig 4A). Combination of OMVs with the IKK inhibitor reduced the bacterial load to 50% at 48 h p.i., compared to DMSO control. To test whether NF-κB inhibition also affected the expression of M1/M2 markers, qPCR analysis was performed. For all analyzed M1 markers, the expression was reduced at the 0 h time point, *i*.*e*. by OMV pre-treatment (Fig 4B–4D). This reduction was not maintained throughout the course of infection in any of the observed cases. CD206, an M2 marker, showed an increased expression when NF-κB was inhibited (Fig 4E).

This implies that the canonical NF-κB pathway is necessary for early M1 activation of macrophages and intracellular long-term replication of *L*. *pneumophila*.

### OMV treatment increases cell viability of *L*. *pneumophila* infected macrophages

Since we observed a strong dependence on NF-κB signaling in the response of macrophages to *L*. *pneumophila* OMVs and since NF-κB has also anti-apoptotic targets [40], we analyzed the influence of OMVs on THP-1 cell viability in the course of infection with *L*. *pneumophila*. The amount of viable cells decreased during infection in not pre-treated cells (24 h p.i.: 16% reduction; 48 h p.i.: 45% reduction in comparison to untreated control cells) as a replication cycle of *L*. *pneumophila* in eukaryotic cells ends with the induction of host cell apoptosis concomitant with bacterial egress [41] (S7A Fig). However, in OMV pre-treated cells, the amount of viable cells was doubled at the lowest dose of OMVs and tripled at the highest dose at 48 h p.i.. We conclude that *L*. *pneumophila* OMVs improve cell viability throughout a following infection with *L*. *pneumophila*.

We investigated the expression of BCL2A1, an anti-apoptotic NF-κB target gene that has been demonstrated to be expressed in *L*. *pneumophila* infection [42]. We observed increased expression of BCL2A1 in OMV pre-treated cells compared to unstimulated cells (S7B Fig). At 24 h p.i., cells treated with higher doses of OMVs still had significantly more BCL2A1 compared to not pre-treated cells. The gain of OMV-treated cells over untreated cells was lost at 48 h p.i., when both pre-treated and not pre-treated cells had a high BCL2A1 expression.

Next we examined whether the activated anti-apoptotic signaling is responsible for the increase in bacterial replication after OMV pre-stimulation of macrophages. To this end, THP-1 cells were pre-treated with the pan-caspase inhibitor zVAD-fmk and then infected with *L*. *pneumophila*. The CFU assay did not reveal significant differences in bacterial replication after caspase inhibition (S7C Fig).

The expression of BCL2A1 together with a higher level of cell viability suggests that OMV exposure activates anti-apoptotic signaling via NF-κB in macrophages.

### *L*. *pneumophila* OMVs lead to miR-146a induction

**Fig 5:**
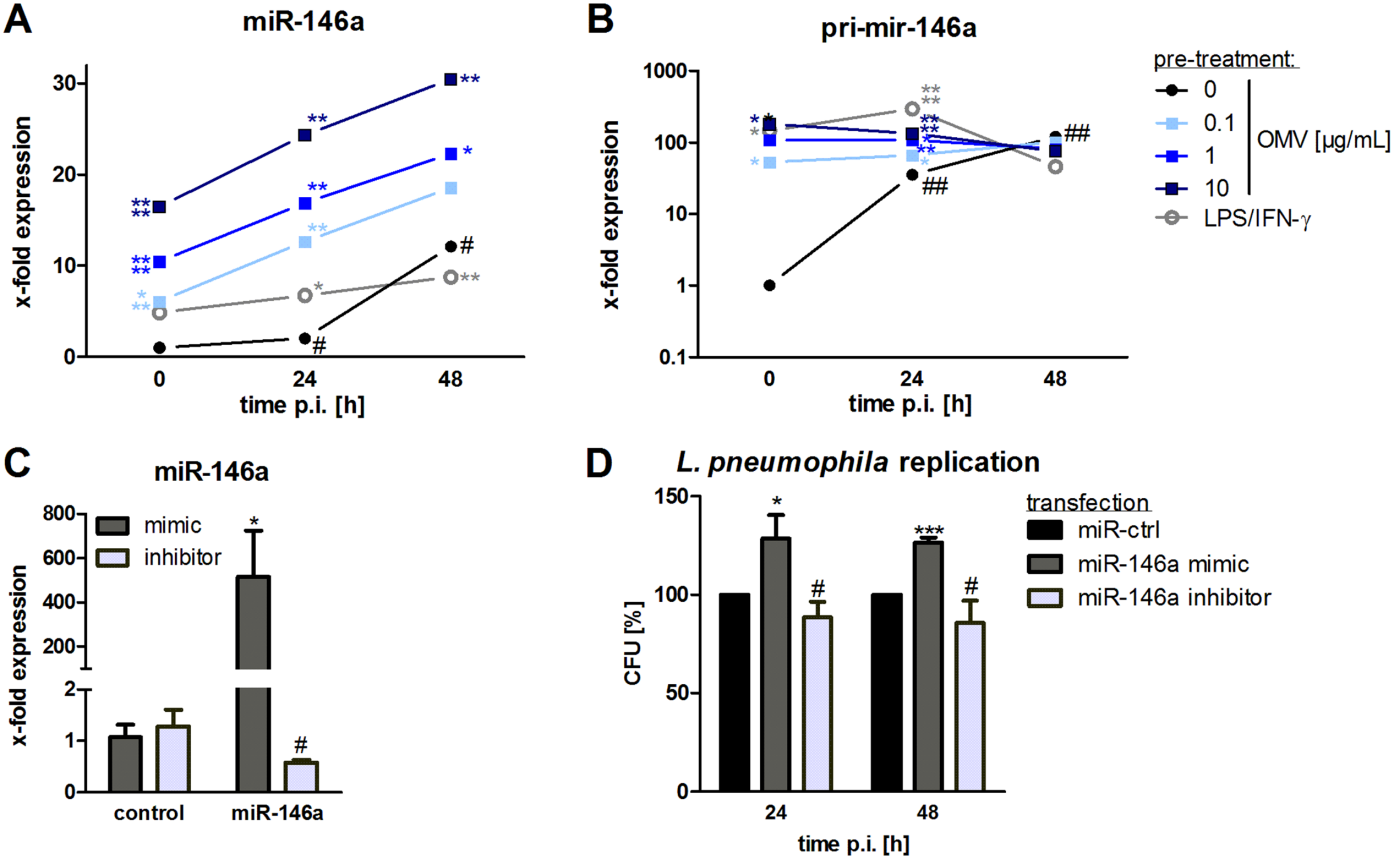
OMVs induce miR-146a upregulation which augments bacterial replication. (A+B) THP-1 cells were pre-incubated with OMVs (0.1, 1, 10 μg/mL) or LPS/IFN-γ for 20 h and then infected with *L*. *pneumophila* (MOI 0.5) for 24 h and 48 h, respectively. qPCR was performed for miR-146a (A) and pri-mir-146a (B). Mean values of three independent experiments are shown. (C+D) THP-1 cells were transfected with miR-146a mimic or inhibitor and corresponding controls. After 24 h of transfection, cells were PMA-differentiated and RNA samples were taken after 24 h incubation. qPCR for miR-146a was performed (C). (D) Transfected THP-1 cells were then additionally infected with *L*. *pneumophila* (MOI 0.5) and lysed for CFU 24 and 48 h p.i., respectively. Results are depicted relative to control transfected cells. Mean values of three independent experiments are shown. Statistics: A+B: Mann-Whitney test; *p<0.05, **p<0.01, ***p<0.001, ****p<0.0001 compared to corresponding 0 μg/mL OMVs. # p<0.05 compared to equally treated 0 h sample. C+D: Mann-Whitney test; *p<0.05, ***p<0.001 compared to corresponding control transfected sample. # p<0.05 compared to miR-146a mimic transfection.

The strong dependence on intact NF-κB signaling for *L*. *pneumophila* replication and the involvement of TLR2 in OMV recognition led us to test for expression of the anti-inflammatory microRNA 146a (miR-146a), as it has been shown to be involved in the response to infections [43]. After OMV treatment of THP-1 cells, we observed a dose-dependent increase in miR-146a expression (Fig 5A). LPS/IFN-γ treatment also led to an induction of miR-146a, albeit to a weaker extent. When cells were additionally infected with *L*. *pneumophila*, the expression of miR-146a further increased over the time. Of note, *L*. *pneumophila* infected cells, which were not pre-treated with OMVs, showed a lower miR-146a expression. The induction of miR-146a could be reduced by blocking TLR2- and NF-κB-signaling (S8A Fig; 69% reduction and 48% reduction). Furthermore, WT mBMDM showed an induction of miR-146a after OMV stimulation (S8B Fig), which was significantly lower in TLR2^-/-^ (35% less miR-146a). TRIF/MyD88 double knockouts did not express miR-146a after OMV stimulation.

The expression of the primary transcript of miR-146a (pri-mir-146a) is NF-κB dependent [44]. As we did not observe a further transcriptional induction of the M1 markers following *Legionella* infection of OMV pre-treated THP-1 cells (Fig 3), we asked whether the strong induction of miR-146a was due to transcriptional induction or processing of pri-mir-146a. OMV treatment of THP-1 cells strongly induced the primary transcript, which could not further be induced by *L*. *pneumophila* infection (Fig 5B). The not pre-treated cells showed a transcriptional induction of pri-mir-146a in response to *L*. *pneumophila* infection. Thus, transcriptional activation, rather than processing, seems to be responsible for the observed elevated miR-146a levels upon infection. OMV pre-treatment already induced the primary transcript of miR-146a that is then processed following *L*. *pneumophila* infection, leading to further increase of the mature miRNA.

The strong expression of miR-146a after OMV stimulation, concomitant with the dependence on active NF-κB signaling, suggest that the TLR-NF-κB pathway may play a role in the macrophage response to *L*. *pneumophila* OMVs. It has been shown that mycobacterial replication in macrophages is facilitated by miR-146a [45]. As we observed an increase in *L*. *pneumophila* replication following OMV stimulation together with an increase in miR-146a, we transfected miR-146a mimic/inhibitor or a corresponding control and infected the cells with *L*. *pneumophila*. Overexpression of miR-146a (Fig 5C) resulted in a 20% enhanced *L*. *pneumophila* number 24 h p.i. which was maintained throughout the experiment (Fig 5D). On the contrary, THP-1 cells transfected with miR-146a inhibitor had significantly less *L*. *pneumophila* replication than miR-146a overexpressing cells (24 h p.i.: 40%, 48 h p.i.: 41%; Fig 5D), arguing for an involvement of miR-146a in the response of macrophages to *L*. *pneumophila* OMVs.

### IRAK-1 degradation is important for *L*. *pneumophila* replication after OMV stimulation

**Fig 6:**
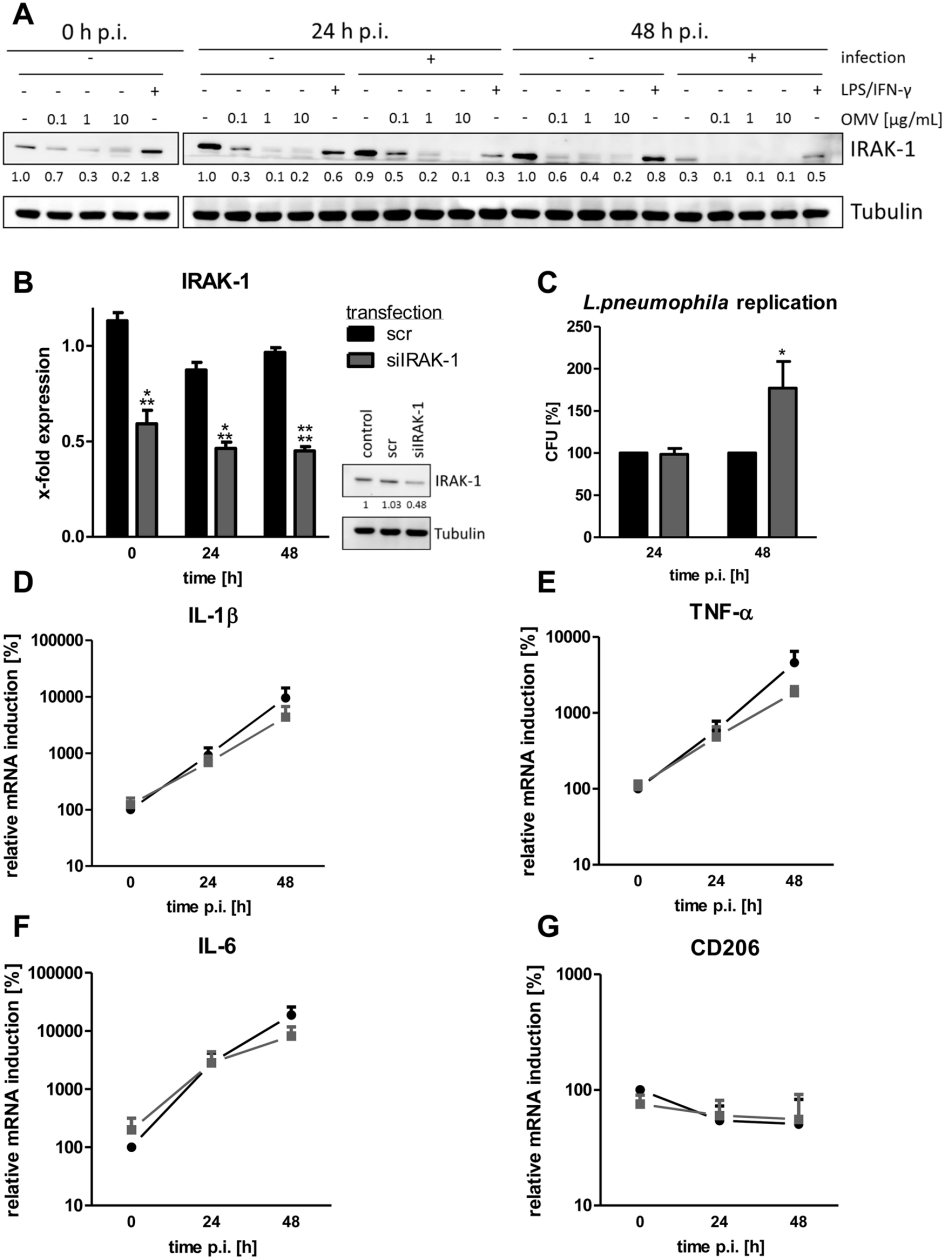
OMVs lead to IRAK-1 degradation and knockdown of IRAK-1 enhances *L*. *pneumophila* replication. A) THP-1 cells were pre-incubated with OMVs (0.1, 1, 10 μg/mL) or LPS/IFN-γ for 20 h and then infected with *L*. *pneumophila* (MOI 0.5) for 24 h and 48 h, respectively. The expression of IRAK-1 was determined by western blot. One representative out of three biological replicates is shown. (B-G) THP-1 cells were transfected with siRNA targeting IRAK-1 or a scramble control. After 24 h of transfection, THP-1 cells were PMA-differentiated. (B) RNA and protein samples were taken after the indicated time points. Expression of IRAK-1 was analyzed by qPCR and western blot. Mean values of four independent experiments are shown for qPCR, one representative out of three biological replicates is shown for western blot. (C) Transfected and PMA-differentiated THP-1 cells were additionally infected with *L*. *pneumophila* (MOI 0.5) for 24 and 48 h and then lysed for CFU assay. Results are depicted relative to scramble control. Mean values of four independent experiments are shown. (D-F) qPCR for markers of classically activated macrophages (IL-1β (D), TNF-α (E), and IL-6 (F)) and alternatively activated macrophages (CD206 (G)) are presented relative to the time point of infection (0 h p.i.) in scramble transfected cells. Mean values of four independent experiments are shown. Statistics: B-G: Mann-Whitney test; **p<0.01, ***p<0.001 compared to corresponding scramble transfected sample.

One well-described target of miR-146a is the kinase IRAK-1 [43, 46]. Upon TLR2 activation, IRAK-1 mediates downstream signaling, undergoing phosphorylation, polyubiquitination and subsequent degradation [47]. Its 3’ UTR can be bound by miR-146a, suppressing its translation. We analyzed IRAK-1 protein expression in OMV stimulated THP-1 cells and observed a strong reduction in IRAK-1 expression after incubation with *L*. *pneumophila* OMVs (Fig 6A). The infection of OMV pre-treated cells further decreased IRAK-1 protein levels more than *L*. *pneumophila* infection alone. In contrast, combination of LPS and IFN-γ led to an increase of IRAK-1 on protein (Fig 6A). Furthermore, LPS/IFN-γ pre-treated cells showed a higher IRAK-1 protein expression than OMV pre-treated cells following infection. Quantification of western blots from three independent experiments is shown in S9A and S9B Fig.

As THP-1 cells responded differently to LTA or LPS as a first stimulus before infection with *L*. *pneumophila*, we analyzed IRAK-1 protein expression after treatment with these TLR ligands. The stimulation of THP-1 cells with LPS for 20 h led to the degradation of 19% of IRAK-1 protein and LTA reduced the IRAK-1 protein level by 17% (S9C and S9D Fig). In contrast, 10 μg/mL OMVs led to the degradation of 70% IRAK-1 protein, which was maintained throughout the incubation.

As a prolonged absence of IRAK-1 protein seems to be favorable for *L*. *pneumophila* replication, knockdown experiments for IRAK-1 were performed. The siRNA-mediated knockdown of IRAK-1 led to 50% reduced mRNA and protein levels in comparison to scramble (scr) transfected control cells at the time point of infection (Fig 6B), which remained stable during the time course of the infection (24 h p.i.: 57% reduction; 48 h: 57% reduction). At 24 h p.i., there was no difference in *L*. *pneumophila* replication compared to scramble control, whereas at 48 h p.i., there was 75% more *L*. *pneumophila* replication in IRAK-1 silenced cells (Fig 6C).

As IRAK-1 is an essential signaling molecule in TLR/IL-1R signaling, the expression levels of macrophage markers were measured after IRAK-1 silencing. THP-1 cells transfected with siIRAK-1 and infected with *L*. *pneumophila* showed less expression of markers for classically activated macrophages (IL-1β, IL-6, and TNF-α) in comparison to scramble control at 48 h p.i. (Fig 6D–6F), whereas the STAT6-dependent CD206, a marker for alternative activation, remained unchanged (Fig 6G).

These experiments show an involvement of IRAK-1 in the response of macrophages to *L*. *pneumophila* OMVs and that a decrease of IRAK-1 protein by OMV pre-treatment was associated with enhanced bacterial replication.

## Discussion

OMVs are potent pro-inflammatory stimulators of different cell types, carrying endotoxin and additional bacterial antigens [4]. OMVs from *Acinetobacter baumannii* induce cytokine secretion in epithelial cells [48], *Clostridium perfringens* OMVs stimulate the murine macrophage cell line RAW264.7 to produce G-CSF, TNF-α, and IL-6 [6]. *L*. *pneumophila* also produces OMVs that can be taken up by macrophages and furthermore induce tissue damage in human lung tissue explants [23]. Until now, the majority of OMV studies focused on direct OMV-mediated changes in their target cell/tissue. In this study, we investigated the impact of OMV pre-treatment on a following encounter of macrophages with *L*. *pneumophila*. THP-1 cells responded upwards of 0.01 μg/mL OMV with IL-8 secretion in a time- and dose-dependent manner. Additionally, the cells secreted IL-1β, IL-6, IL-10, and TNF-α dose-dependently. In contrast to THP-1 cells, stimulation of an alveolar type II epithelial cell line with *L*. *pneumophila* OMVs was reported to require much higher doses to induce pro-inflammatory activation (50 μg in contrast to 0.01 μg/mL) [22]. Human primary macrophages induce TNF-α release when exposed to 0.3 μg/mL *L*. *pneumophila* OMVs [24]. Here, we observed a response intensity to OMVs similar to what others observed when administering whole *L*. *pneumophila* [42]. Based on LPS-measurements, we assume that the experimentally used OMV doses resemble those that occur under infection conditions.

It has been described that LPS is present on the OMV surface [27, 28]. Therefore, we analyzed the importance of TLR signaling in macrophage activation by OMVs. mBMDMs from TLR2/4^-/-^ mice showed a reduced CXCL1 secretion upon OMV exposure in comparison to cells from wildtype mice. Our results are in line with the results obtained by Jäger *et al*., who performed experiments with HEK293 cells overexpressing either TLR2 or TLR4 and observed a stronger response to OMVs when TLR2 was overexpressed [24]. Hence, LPS might be the most important stimulant on OMVs derived from *L*. *pneumophila*.

The two most important OMV components, LPS and proteins, are both essential for the initial activation of macrophages. The combination of both and the physiological context in which they are recognized seem to determine their effect on the host. This has recently been demonstrated for *Pseudomonas aeruginosa* OMVs [5]. Likewise, knockout mice for either TLR2 or TLR4 still show induction of inflammation in the lungs after being treated with *P*. *aeruginosa* OMVs [49].

Since *L*. *pneumophila* OMVs can contain the TLR5-activating fliC [22, 35], we pre-exposed THP-1 cells to OMVs obtained from ΔflaA *L*. *pneumophila*, but no differences could be observed in comparison to OMVs from wildtype *L*. *pneumophila*.

The infection of THP-1 with *L*. *pneumophila* led to an increased expression of classical (M1) macrophage activation markers. However, upon pre-exposure to OMVs, virtually no further increase was observed upon infection with viable bacteria. A reduced macrophage response to classical TLR stimuli has also been observed in a study with *Brucella abortus* OMVs, where THP-1 cells secreted less TNF-α and IL-8 in response to LPS, Pam3Cys and flagellin after OMV challenge [25]. Furthermore, *Porphyromonas gingivalis* OMVs are also reducing the TNF-α response to a second LPS stimulus [26]. We studied the impact of OMVs on a subsequent infection. We did not observe any differences in bacterial uptake capacity. However, 24 h post infection, OMV pre-treatment led to a reduction of bacterial replication in a similar range as LPS/IFN-γ pre-exposure. LPS/IFN-γ is an inducer of the M1 phenotype, which is capable of enhanced bacterial killing [29]. This restrictive effect on *Legionella* replication seems to depend in part on both OMV-transported proteins and TLR2 ligands, as it could be neutralized by heat exposure or treatment with a TLR2 blocking antibody, and mimicked by TLR2 activation. In addition, the early growth restriction could be reverted by inhibition of the canonical NF-κB pathway, because *L*. *pneumophila* needs the transcriptional induction of anti-apoptotic NF-κB target genes [42, 50]. In contrast to the early time point of infection (24 h p.i.), we observed a boosting effect of OMV pre-treatment on bacterial replication at 48 h time point, both in THP-1 cells and primary mBMDM of wildtype mice. Induction of bacterial replication after OMV treatment has also been observed in a study with *Mycobacterium bovis* in mice [51]. There, challenging mice with OMVs prior to an infection led to a doubling in bacterial load in the lungs and more spreading of the bacteria to the spleen. In contrast to the early restrictive effect on *Legionella* replication, the subsequently observed boost in replication seems not to rely on proteins or nucleic acids transported by OMVs and could not be mimicked by LPS or LTA preparations. However, the OMV-induced increase in *L*. *pneumophila* replication at the later time point was completely abolished by TLR2 knockout, TRIF/MyD88 double knockout, or inhibition of the canonical NF-κB pathway. Interestingly, OMV pre-treatment of macrophages before the infection with a dot/icm mutant of *Legionella* (ΔdotA), which is not capable of forming a LCV under normal conditions [17, 30], enables this mutant to replicate in THP-1 cells. Furthermore, the increased *L*. *pneumophila* replication was lost when OMVs were incubated with a membrane-permeabilizing agent, implicating that not only LPS on the OMV surface is critical for the observed effect, but also the context in which it is presented. Ellis *et al*. made similar observations with OMVs from *P*. *aeruginosa* [5]. LPS, which is important for binding of the OMV to the macrophage surface, or proteins, which are required for the internalization, are not sufficient alone. The observation that LPS aggregate size influences its internalization [52] and that vesicular LPS has a higher potency to activate macrophages [5], supports the concept that the three-dimensional structure of the vesicles impacts the response. Our results support the model that the response of inflammatory cells depends of the context in which LPS is presented [53].

*L*. *pneumophila* critically depends on establishing a fine balance between avoiding the immune response and successful infection. Pro-inflammatory cytokines and anti-apoptotic proteins are equally induced after *L*. *pneumophila* infection and are both dependent on p65 translocation into the nucleus [35, 42], which we observed after short time of OMV incubation. *L*. *pneumophila* itself can establish a phosphorylation of IκBα at ser-52 and ser-36 by LnaB and LegK1 which mimic eukaryotic serine/threonine kinases; this again leads to an IKK independent and robust induction of apoptosis antagonist genes as well as pro-inflammatory genes [54, 55]. As *L*. *pneumophila* activates NF-κB signaling itself and critically depends on this signaling as demonstrated by CFU assay with the IKK inhibitor, we conclude that macrophages might be a better replication niche for *L*. *pneumophila* when NF-κB signaling has already been induced by a first stimulus. There was no complete loss of bacterial replication observed upon IKK-inhibitor treatment, since SAPK/JNK and p38 signaling are still intact and also support *L*. *pneumophila* replication [56].

At a late time point during infection, OMV pre-treated cells showed better survival than not pre-treated macrophages. This might be a result of the activation of anti-apoptotic signaling via NF-κB, as we found increased BCL2A1 expression after OMV exposure. This BCL2 family member can be induced by GM-CSF, LPS, or TNF-α [57–59] via the transcription factor NF-κB [60]. The expression of anti-apoptotic BCL2A1 is in line with the survival advantage of OMV-treated cells when infected with *L*. *pneumophila*. However, caspase inhibition did not alter bacterial replication.

The *L*. *pneumophila* replication cycle in eukaryotic cells ends with host cell apoptosis and bacterial egress [41, 61], but here we observed an increase in bacterial replication after OMV incubation concomitant with a higher rate of cell viability. In addition to this, we found that OMV pre-treatment led to an increased number of vacuoles per cell, suggesting that exposure to OMVs rendered human macrophages more susceptible to intracellular *L*. *pneumophila* replication.

TLR and NF-κB signaling are tightly controlled not only by posttranslational modifications of involved proteins, but also by miRNAs that act on the 3’ UTR of mRNAs and lead to their translational repression [62]. *L*. *pneumophila* OMVs increase the levels of miR-146a by induction of its primary transcript, pri-mir-146a via TLR2 and NF-κB. This anti-inflammatory miRNA is involved in the intracellular replication of *Mycobacterium bovis* in macrophages [45]. We show that the artificial overexpression of miR-146a prior to *L*. *pneumophila* infection results in enhanced bacterial replication whereas the knockdown of this miRNA decreases the replication. This loss-of-function effect was not very strong in CFU assay, presumably because a functional knockdown of a microRNA as highly abundant as miR-146a is difficult to achieve. In addition, we are measuring a sum signal of only partly transfected cells. Moreover, the transfection of microRNAs can alter the loading of endogenous miRNAs into the microRNA processing machinery [63] and this could influence mRNA targets that we did not investigate.

The effect on bacterial replication of miR-146a in *M*. *bovis* infection has been linked to IRAK-1, which is a well-studied target of this miRNA [45]. IRAK-1 is essential in TLR and IL-1R signaling, as it mediates the downstream activation of NF-κB [47]. As we observed a long lasting IRAK-1 simultaneously with p65 nuclear translocation, we hypothesized that it is involved in the replication enhancing effect observed by OMV pre-treatment of macrophages. Knockdown of IRAK-1 by siRNA increased *L*. *pneumophila* replication and mimicked the effect observed with OMVs. Exposure of THP-1 cells to OMVs led to stronger effects on *L*. *pneumophila* replication than IRAK-1 knockdown, probably due to the moderate knockdown efficiency. Markers for classical macrophage activation were also reduced in siIRAK-1 transfected cells, similarly to OMV pre-treatment. It has been previously shown that LPS exposure can desensitize cells for a second LPS challenge by miR-146a upregulation, IRAK-1 degradation and translational repression of IRAK1 mRNA, which then results in a reduced cytokine response to the second LPS stimulus [64]. TLR2 or TLR4 activation alone did not mimic the full repertoire of *L*. *pneumophila* OMV exposure, probably because it results in much higher remaining IRAK-1 protein levels than OMV pre-treatment, even though the LPS doses were comparable (200 ng/ml *S*. *minnesota* LPS was used and 1 μg/mL OMV contains 220 ng/mL LPS). Contrasting OMV treatment, LPS/IFN-γ even induced IRAK-1 on protein level and led to reduced *L*. *pneumophila* replication in THP-1 cells.

Our results and the cited studies point towards an immunomodulatory action of OMVs, which might help the bacteria evading the host immune system. To our knowledge, our report is the first to describe the phenomenon of an OMV-induced replication advantage of *L*. *pneumophila*. *Legionella* has developed several sophisticated strategies to ensure its replication and spreading by evading the host immune system. They hijack key cellular processes to avoid lysosomal degradation after phagocytosis, and they interfere with host vesicular trafficking, ubiquitination and autophagy [17, 65, 66]. In doing so, *L*. *pneumophila* gains control over the host innate immune response. The activation of NF-κB signaling, by either OMVs or whole bacteria, leads to secretion of pro-inflammatory cytokines, which likely results in the recruitment of new potential host cells to the site of infection, which then can be infected. The increased infection of macrophages in the presence of OMVs might therefore be an important pathogenic strategy of *L*. *pneumophila*.

**Fig 7:**
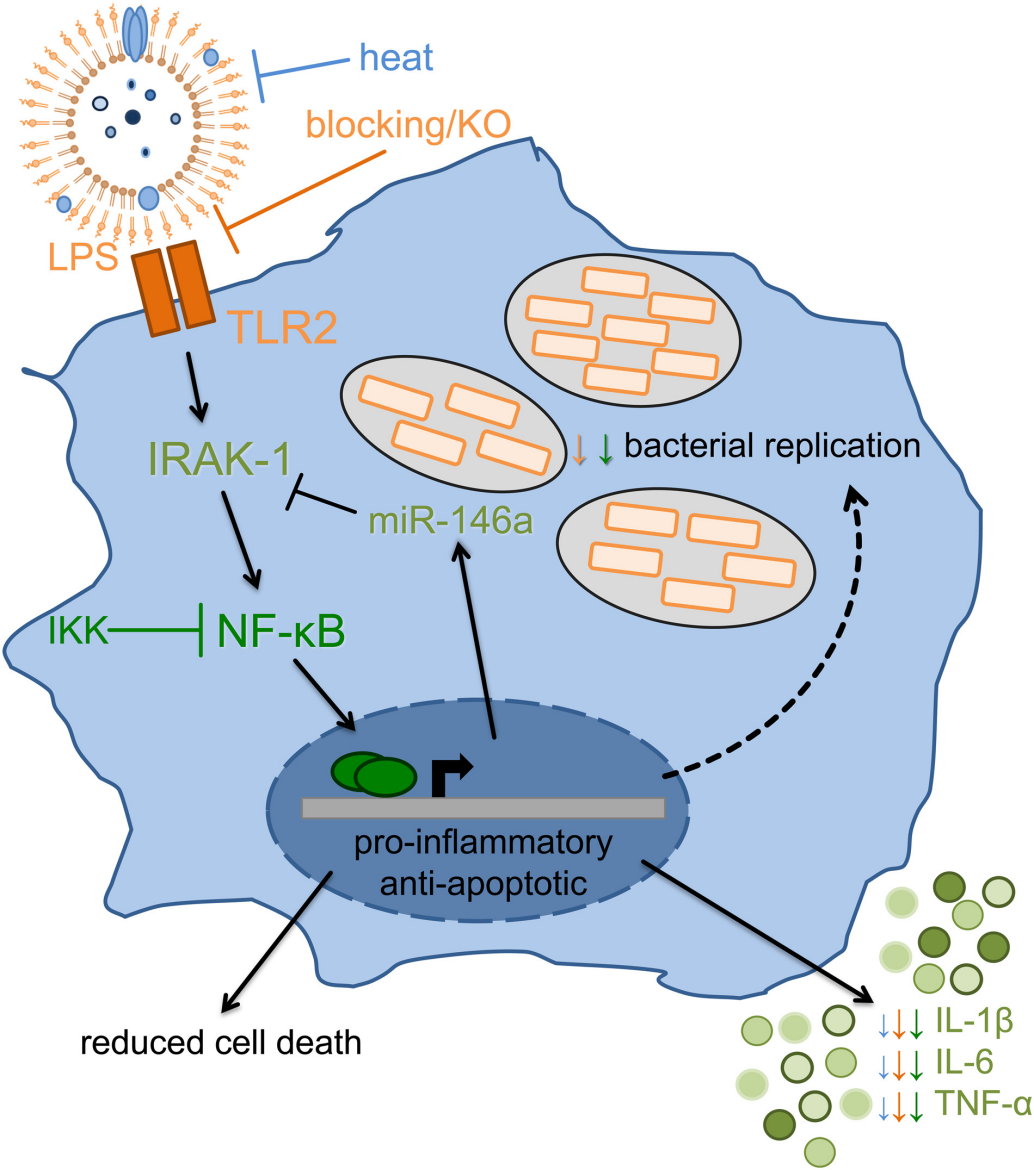
Response of macrophages to OMVs. Macrophages sense *L*. *pneumophila* OMVs via TLR2, which is critical for the initial activation of macrophages, and which results in IRAK-1 degradation. The downstream NF-κB nuclear translocation and signaling is essential for the induction of cytokine gene expression and miR-146a induction. A subsequent infection of OMV pre-treated macrophages with *Legionella* results in a higher rate of bacterial replication concurrent with more vacuoles per cell. While pro-inflammatory gene expression caused by OMVs cannot be further enhanced by *L*. *pneumophila* infection, there is induced transcription of anti-apoptotic genes. This results in increased viability of infected cells. Prolonged IRAK-1 absence, via protein degradation and suppressed translation by miR-146a, leads to a more efficient replication of *L*. *pneumophila*.

Taken together, this study has demonstrated for the first time that OMVs directly influence the course of *Legionella* infection by activating macrophages in a pro-inflammatory way and at the same time promoting bacterial replication (Fig 7). This property seems to depend on TLR2- and NF-κB-dependent miR-146a upregulation and consequently prolonged IRAK-1 depletion. Thereby, OMVs could facilitate replication and spreading of *L*. *pneumophila* in the human lung.

## Materials and Methods

### Chemicals and antibodies

RPMI-1640, FCS, phalloidin Alexa Fluor 546 and goat anti-mouse Alex Fluor 488 were obtained from LifeTechnologies (Darmstadt, Germany). PBS was from Biochrom GmbH (Berlin, Germany). PMA was purchased from Sigma-Aldrich Chemie GmbH (Taufkirchen, Germany). LTA was from InvivoGen (Toulouse, France). DAPI was from AAT Bioquest (Sunnyvale, USA). IKK inhibitor XIII was obtained from EMD Millipore Corporation (Billerica, USA). zVAD-fmk was obtained from AdipoGen (Liestal, Switzerland) *Salmonella minnesota* LPS was from Enzo Life Sciences (Lörrach, Germany). Human recombinant IFN-γ was obtained from PromoKine (Heidelberg, Germany). TLR2 blocking antibody was acquired from eBioscience (T2.5, San Diego, USA). Anti-LPS antibody (*L*. *pneumophila* specific; ABIN235748) was obtained from antikörper-online.de (Aachen, Germany). Anti-IRAK-1 antibody was purchased from Cell Signaling (4359S), anti-tubulin antibody was from Santa Cruz (sc-5286) as well as p65 antibody (sc-372) and β-actin antibody (sc-1616). All chemicals used were of analytical grade and obtained from commercial sources.

### *L*. *pneumophila* culture and OMV preparation

*Legionella pneumophila* strain Corby wildtype was grown on buffered charcoal-yeast extract (BCYE) agar at 37°C for three days. For OMV preparation, *L*. *pneumophila* were transferred from BCYE agar plates into sterile yeast extract broth (YEB) medium at a density of 1x10^9^ bacteria per mL. Bacteria were incubated in a shaking incubator (MaxQ 6000, Thermo Fisher Scientific, Karlsruhe, Germany) at 37°C until they reached early stationary phase. Pure *L*. *pneumophila* cultures were then spun down three times (4,500 x g, 15 min, 4°C; Multifuge X3R, Thermo Fisher Scientific). To remove remaining bacteria, the supernatant was passed twice through a 0.22 μm sterile filter. The supernatant was then ultracentrifuged (100,000 x g, 3 h, 4°C). After washing the obtained OMV pellet with sterile PBS to remove contaminating free protein, it was again ultracentrifuged and then resuspended in sterile PBS and stored at -20°C. The protein content of the preparations was determined by Pierce BCA protein assay kit (Thermo Fisher Scientific), and equal protein amounts were used for cell stimulation. For protein denaturation, OMVs were incubated at 60°C for 5 min as previously described [33].

### Cell culture and *L*. *pneumophila* infection

The human monocytic cell line THP-1 was obtained from American Type Culture Collection (Rockville, MD, USA) and cultivated in RPMI-1640 with supplements and 10% FCS in a humidified incubator at 37°C and 5% CO_2_. THP-1 cells were differentiated into a macrophage-like phenotype using 20 nM PMA for 24 h and then plated at the desired density. For all infection experiments, differentiated THP-1 cells were infected with WT *L*. *pneumophila* with a multiplicity of infection (MOI) of 0.5. For infection experiments of mBMDM *L*. *pneumophila* ΔflaA were used at an MOI of 0.5.

Murine bone marrow derived monocytes were isolated from tibiae and femora of wildytpe, TLR2^-/-^, TLR2/4^-/-^ or TRIF/MyD88^-/-^ mice (C57BL/6N). For each experiment, cells were differentiated into macrophages in the presence of M-CSF (macrophage colony-stimulating factor). After 72 h of incubation, M-CSF was added again, and cells were incubated for additional 48 h. Differentiated cells were detached and re-plated at the desired density in the presence of GM-CSF. After 24 h, cells were used for experiments.

### Transfection of THP-1 cells

Transfection of THP-1 cells with miRNA mimics or inhibitors was performed with siPORT NeoFX (Invitrogen) according to the manufacturer’s instructions. mirVana mimics and inhibitors for miR-146a and corresponding controls were purchased from Thermo Fisher Scientific. Following the manufacturer’s protocol, siRNA transfection was performed by using Lipofectamine 2000 (Thermo Fisher Scientific). Silencer select siRNA targeting IRAK-1 mRNA and a corresponding scramble control were purchased from Thermo Fisher Scientific.

### OMV stimulation of macrophages

Differentiated THP-1 or mBMDM were stimulated with different doses of OMVs (0.01–25 μg/mL as determined by the protein concentration of the OMV preparation). Cells were incubated with OMVs for 24 or 48 h. In all infection experiments OMV pre-incubation was carried out for 20 h and then the infection followed for up to 48 h. LPS (200 ng/mL) alone or in combination with IFN-γ (200 ng/mL) or LTA (1 μg/mL) were alternatively used to pre-stimulate THP-1 cells for 20 h before the infection with *L*. *pneumophila*. To assess the influence of TLR2 signaling, a TLR2 blocking antibody was added to the cells 90 min before OMV stimulation at a final concentration of 20 μg/mL. NF-κB inhibition was achieved by incubation with 1 μM IKK XIII inhibitor prior to treatment. The influence of nucleic acids in the OMVs on *L*. *pneumophila* replication was analyzed by digestion of OMVs with RNase A (0.2 μg/μL) and RNase III (0.02 U/μL) in combination or DNase I (0.004 U/μL). OMVs were incubated for 1 h at 37°C with the enzymes alone or in combination with 0.3% Triton X-100 to permeabilize the OMV membrane (final concentration in the cell culture well: 0.75 ‰).

### Colony forming unit assay (CFU)

To analyze bacterial replication in THP-1 cells, cells were pre-stimulated with rising doses of OMVs or left untreated and subsequently infected with *L*. *pneumophila*. To quantify the bacterial load, cells were lysed at indicated time points with saponin (0.1% in the supernatant) and different dilutions of the lysates were streaked on BCYE agar plates. After three days of incubation at 37°C, *L*. *pneumophila* colonies were counted and the bacterial load was calculated.

### ELISA

IL-8 or CXCL1 from the cell-free supernatant of THP-1 or mBMDM was analyzed with commercial ELISA kits (IL-8: OptEIA, BD Biosciences, Heidelberg, Germany; CXCL1: DuoSet, R&D, Minneapolis, USA). All other secreted cytokines (IL-1β, IL-6, IL-10, TNF-α, MCP-1, GM-CSF) were measured with a Luminex Assay (R&D) in a Bio-Plex Magpix (Luminex Corporation, Austin, USA) according to the manufacturer’s instructions.

### RNA preparation and real-time PCR

For analysis of gene expression, total RNA isolation was carried out by phenol-chloroform extraction with Isol RNA lysis reagent (5Prime, Hamburg, Germany). For the detection of pri-mirnas, purified RNA was DNase I digested (Roche, Mannheim, Germany). After reverse transcription (High-Capacity RNA-to-cDNA kit or TaqMan miRNA reverse transcription kit, both Thermo Fisher Scientific), quantitative real-time PCR was performed in a ViiA7 (Thermo Fisher Scientific) with Fast SYBR Green Master Mix (Thermo Fisher Scientific) and specific primer pairs. miRNA expression was analyzed with TaqMan assays detecting miR-146a, pri-mir-146a, pri-mir-16-2 and RNU48 (Thermo Fisher Scientific).

BCL2A1: fwd: 5’-GGCCCACAAGAAGAGGAAAATG-3’, rev: 5’-GGAGTGTCCTTTCTGGTCAACA-3’

CD206: fwd: 5‘-CAGCGCTTGTGATCTTCATT-3‘, rev: 5‘-TACCCCTGCTCCTGGTTTTT-3‘

IL-1β: fwd: 5‘-AGCTCGCCAGTGAAATGATGG-3‘, rev: 5‘-CAGGTCCTGGAAGGAGCACTTC-3‘

IL-6: fwd: 5‘-AATTCGGTACATCCTCGACGG-3‘, rev: 5‘-TTGGAAGGTTCAGGTTGTTTTCT-3‘

IRAK-1: fwd: 5’-TGAGGAACACGGTGTATGCTG-3‘, rev: 5‘-GTTTGGGTGACGAAACCTGGA-3’

RPS18: fwd: 5’-GCGGCGGAAAATAGCCTTTG-3‘, rev: 5‘-GATCACACGTTCCACCTCATC-3‘

TNF-α: fwd: 5‘-GCTGCACTTTGGAGTGATCG-3‘, rev: 5’-TCACTCGGGGTTCGAGAAGA-3‘

### MTT

Cells were seeded into microtiter plates and incubated with rising amounts of OMVs before infection with *L*. *pneumophila*. At indicated time points, 10 μL thiazolyl blue tetrazolium bromide (MTT, 5 mg/mL; Sigma-Aldrich Chemie GmbH) was added to the cells and incubated for another 2 h at 37°C. Medium was removed and replaced with EtOH:DMSO (1:2). After shaking, absorption was measured at 570 nm.

### Immunofluorescence

THP-1 cells were seeded on coverslips and infected with *L*. *pneumophila* for 48 h with or without OMV pre-incubation. After 15 min fixation with 4% paraformaldehyde, slides were washed three times with PBS and permeabilized with 0.2% Triton X-100 in TBS. Blocking was carried out with 10% FCS for 90 min. Incubation with α-LPS (1:500, in blocking solution) was followed by α-mouse (1:1000, in blocking solution) in combination with DAPI (1:2000) and phalloidin Alexa Fluor 546 (1:40). Stainings were analyzed on a fluorescence microscope (Axio Vision, Zeiss, Jena, Germany).

### Ethical statement

All animals were handled according to national and European legislation, namely the EU council directive 86/609/EEC for the protection of animals. The performed protocols were approved by the responsible animal ethics committee (Philipps-University Marburg; permit number: EX-22-2013).

### Statistics

Data are shown as mean values ± SEM for at least three biologically independent experiments. Prism 5 (GraphPad, La Jolla, USA) was used. The non-parametric Mann-Whitney test was performed for unpaired samples. P-values ≤ 0.05 were considered statistically significant. If not indicated otherwise, tests were performed vs. corresponding control (*).

## Supporting Information

S1 Fig Cell viability after OMV treatment. THP-1 cells were treated with different doses of OMVs (0.1, 1, 10 μg/mL) and incubated for up to 96 h. Cell viability was determined by MTT assay and results are normalized to untreated control cells at every time point. Results are presented as mean values of four independent experiments, each performed in technical triplicates. Statistics: Mann-Whitney test; *p<0.05 compared to 0 μg/mL OMVs.
(TIF)

S2 Fig *L*. *pneumophila* replication in THP-1 cells. (A) THP-1 cells were infected with *L*. *pneumophila* at an MOI of 0.5 and bacterial replication was determined by CFU assay. Cells were lysed after the indicated time points. Shown are mean values of three independent experiments each performed in duplicates. (B) *L*. *pneumophila* uptake in THP-1 cells after pre-treatment with OMVs (0.1, 1 or 10 μg/mL) or LPS/IFN-γ is shown by CFU assay; not pre-treated cells served as a control. Shown are mean values of three independent experiments, each performed in duplicates. (C) Bacterial replication of *L*. *pneumophila* ΔdotA was analyzed in THP-1 cells with OMV pre-treatment (WT *L*. *pneumophila*) by CFU assay 24 and 48 h p.i.. Results are depicted relative to CFU count of not pre-treated (but infected) cells. Shown are mean values of three independent experiments, each performed in duplicates. Statistics: Mann-Whitney test; *p<0.05 compared to 0 μg/mL OMVs, ns = not significant.
(TIF)

S3 Fig Influence of OMV proteins and TLR2 activation on *L*. *pneumophila* replication. (A) THP-1 cells were pre-incubated with heat inactivated and intact OMVs (1 μg/mL each) 20 h before infection with *L*. *pneumophila* (MOI 0.5). Bacterial replication was determined by CFU assay. Results are depicted relative to CFU count of not pre-treated (but infected) cells. Mean values of three independent experiments are shown. (B-F) THP-1 cells were pre-incubated with TLR2 blocking antibody or a control (20 μg/mL each) 90 min before OMV stimulation (1 μg/mL). After 20 h of OMV incubation, cells were infected with *L*. *pneumophila* (MOI 0.5) and lysed for CFU 24 and 48 h p.i. (B). Pre-treatment with LTA (1 μg/mL) or LPS (200 ng/mL) served as a control. Results are depicted relative to CFU count of not pre-treated (but infected) cells. Mean values of three independent experiments, each performed in duplicates, are shown. RNA samples were taken at the time point of infection (0 h) or 24 and 48 h p.i.. qPCR was performed for IL-1β (C), TNF-α (D), IL-6 (E), and CD206 (F). Results are calculated relative to the time point of infection (0 h) with 1 μg/mL OMV treatment. Mean values of three independent experiments are shown. Symbol key from S3B Fig is used also in S3C–S3F Fig. (G) *Legionella* replication was determined by CFU assay in mBMDM. Cells from WT, TLR2^-/-^ and TRIF/MyD88^-/-^ mice were pre-treated with 0.1 μg/mL OMV for 20 h and then infected with *L*. *pneumophila* ΔflaA (MOI 0.5) for 48 h. Bars represent mean values of three biological independent experiments, each performed in duplicates. Statistics: A: Mann-Whitney test; *p<0.05 compared to corresponding 0 μg/mL OMVs. B: Mann-Whitney test; *p<0.05, ****p<0.0001 compared to corresponding 0 μg/mL OMVs. #p<0.05 compared to 1 μg/mL OMVs. C-F: Mann-Whitney test; *p<0.05, **p<0.01, ***p<0.001, ****p<0.0001 compared to corresponding 1 μg/mL OMVs. G: Mann-Whitney test; *p<0.05 compared to WT.
(TIF)

S4 Fig Increased *L*. *pneumophila* replication depends on OMV membrane integrity and not on flagellin. A) THP-1 cells were pre-incubated for 20 h with OMVs (1 μg/mL) from wildtype *Legionella* or *Legionella* lacking flagellin (ΔflaA) and were then infected with *L*. *pneumophila* (MOI 0.5). Bacterial replication after OMV pre-stimulation was determined by CFU assay 24 and 48 h p.i.. Results are depicted relative to CFU count of not pre-treated (but infected) cells. B) THP-1 cells were treated with OMVs (1 μg/mL) which were either untreated or treated with RNase (RNase A and RNase III in combination) or DNase alone, respectively, or in combination with Triton X-100 (0.3%). After 20 h pre-incubation, cells were infected with *L*. *pneumophila* (MOI 0.5) and bacterial replication was assessed by CFU assay 24 and 48 h p.i.. *L*. *pneumophila* replication is presented relative to not pre-treated but infected cells. Bars show mean values of three independent experiments, each performed in duplicates. Statistics: A) Mann-Whitney test; *p<0.05 compared to 0 μg/mL OMVs. B) Mann-Whitney test; *p<0.05 compared to corresponding OMV treated sample.
(TIF)

S5 Fig Nuclear shuttling of p65 after OMV treatment. THP-1 cells were treated with 1 μg/mL OMVs for 30 min or left untreated as a control. Protein extracts from nucleus and cytosol were generated and the localization of p65 was determined by western blot. Lamin C served as a nuclear loading control and β-actin as a cytosolic loading control. One representative result is shown.
(TIF)

S6 Fig Cell viability after IKK inhibitor treatment. THP-1 cells were treated with IKK inhibitor (1 μM) or DMSO as a solvent control 90 min before addition of 1 μg/mL OMVs for 20 h in the indicated samples. Cell viability was determined by MTT assay and results are normalized to 0 μg/ML OMVs. Bars represent mean values of three independent experiments, each performed in triplicates. Statistics: Mann-Whitney test; ns = not significant.
(TIF)

S7 Fig OMV pre-treatment increases cell viability throughout a following infection. (A) THP-1 cells were pre-treated with OMVs (0.1, 1, 10 μg/mL) for 20 h before infection with *L*. *pneumophila* (MOI 0.5). Cell viability was determined by MTT assay and results are normalized to 0 μg/mL OMVs at every time point. Results are shown as mean values of four independent experiments, each performed in technical triplicates. (B) OMV-treated THP-1 cells (0.1, 1, 10 μg/mL) or control cells were infected with *L*. *pneumophila* for 24 or 48 h at an MOI of 0.5, respectively. RNA samples were taken at the time point of infection (0 h) or 24 and 48 h p.i.. qPCR was performed for BCL2A1 and results are normalized to 0 μg/mL OMVs at 0 h p.i.. (C) THP-1 cells were treated with zVAD-fmk (10 μg/mL) or DMSO as a solvent control for 90 min before the infection with *L*. *pneumophila* (MOI 0.5) for 24 and 48 h. Bacterial replication was analyzed by CFU assay. Results are present relative to not pre-treated but infected control cells. Bars represent mean values of four biological independent experiments, each performed in duplicates. Statistics: Mann-Whitney test; *p<0.05, ****p<0.0001 compared to 0 μg/mL OMVs or DMSO; ns = not significant.
(TIF)

S8 Fig miR-146a induction depends on TLR- and NF-κB-signaling. (A) THP-1 cells were pre-treated with TLR2 blocking antibody (20 μg/mL) or IKK inhibitor (1 μM) 90 min before addition of 1 μg/mL OMVs. Cells were infected after 20 h of OMV stimulation for 24 and 48 h. RNA samples were taken at the time point of infection (0 h), and 24 and 48 h p.i. and the expression of miR-146a was analyzed. Bars represent x-fold expression and are normalized to untreated control cells. Shown are mean values of three biological independent experiments. (B) miR-146a expression was analyzed in mBMDM (WT, TLR2^-/-^, TRIF/MyD88^-/-^) after the treatment with 0.1 μg/mL OMVs for 20 h. Graph represents x-fold expression in comparison to untreated control cells. Bars are mean values of three biological independent experiments. Statistics: Mann-Whitney test; *p<0.05, **p<0.01, compared to WT.
(TIF)

S9 Fig Varying IRAK-1 degradation after different treatments. (A-B) Three independent experiments as shown in Fig 6A were quantified and the results normalized to untreated control cells. IRAK-1 degradation after (A) OMV or LPS/IFN-γ stimulation without infection and (B) pre-stimulation plus infection with *L*. *pneumophila* are shown. (C) IRAK-1 degradation is shown after pre-treatment of THP-1 cells with LPS (200 ng/mL) or LTA (1 μg/mL) 20 h before infection with *L*. *pneumophila* (MOI 0.5) at the time point of infection (0 h) and 24 and 48 h p.i. One representative experiment out of three biological independent experiments is shown. (D) Comparison of remaining IRAK-1 protein levels after the different treatments (OMV, LTA, LPS) from S9A and S9C Fig. Mean values of three independent experiments are shown. Statistics: (A-B) Mann-Whitney test; *p<0.05, **p<0.01, ***p<0.001, ****p<0.0001 compared to 0 μg/mL OMVs. #p<0.05 compared to LPS/IFN-γ treated cells.
(TIF)
