# Supplementary figures and images for: Legionella pneumophila-Derived Outer Membrane Vesicles Promote Bacterial Replication in Macrophages
Source: PLoS Pathog. 2016 Apr 22;12(4):e1005592. doi: 10.1371/journal.ppat.1005592 (PMC4841580; doi:10.1371/journal.ppat.1005592)

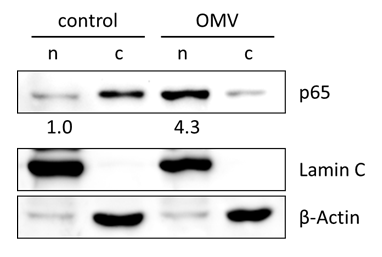

Supplement: S5 Fig — THP-1 cells were treated with 1 μg/mL OMVs for 30 min or left untreated as a control. Protein extracts from nucleus and cytosol were generated and the localization of p65 was determined by western blot. Lamin C served as a nuclear loading control and β-actin as a cytosolic loading control. One representative result is shown. (TIF) [file ppat.1005592.s005.TIF]

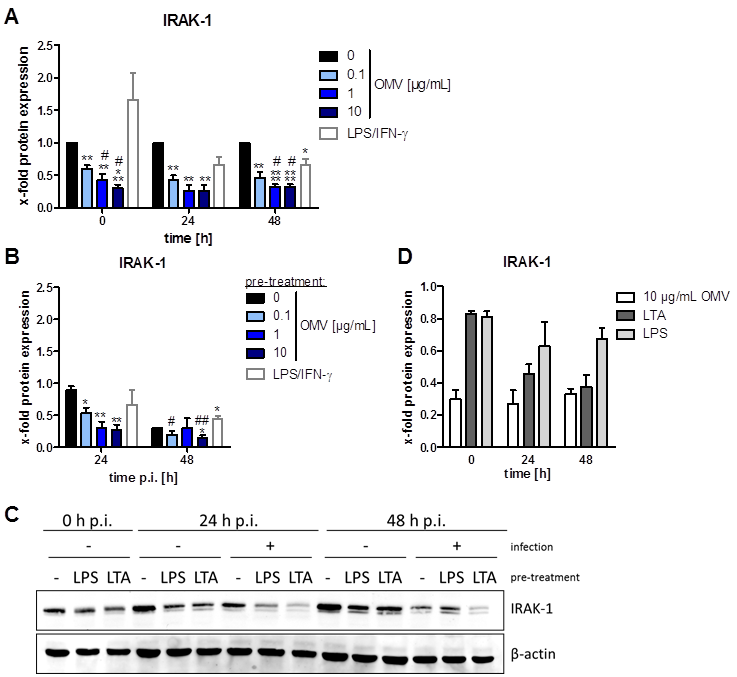

Supplement: S9 Fig — (A-B) Three independent experiments as shown in Fig 6A were quantified and the results normalized to untreated control cells. IRAK-1 degradation after (A) OMV or LPS/IFN-γ stimulation without infection and (B) pre-stimulation plus infection with L. pneumophila are shown. (C) IRAK-1 degradation is shown after pre-treatment of THP-1 cells with LPS (200 ng/mL) or LTA (1 μg/mL) 20 h before infection with L. pneumophila (MOI 0.5) at the time point of infection (0 h) and 24 and 48 h p.i. One representative experiment out of three biological independent experiments is shown. (D) Comparison of remaining IRAK-1 protein levels after the different treatments (OMV, LTA, LPS) from S9A and S9C Fig. Mean values of three independent experiments are shown. Statistics: (A-B) Mann-Whitney test; *p<0.05, **p<0.01, ***p<0.001, ****p<0.0001 compared to 0 μg/mL OMVs. #p<0.05 compared to LPS/IFN-γ treated cells. (TIF) [file ppat.1005592.s009.TIF]
